# NIR-responsive magnesium phosphate cement loaded with simvastatin-nanoparticles with biocompatibility and osteogenesis ability†

**DOI:** 10.1039/d4ra01079e

**Published:** 2024-04-29

**Authors:** Bin Wang, Yanbin Zhao, Yangyang Li, Junyan Yao, Shunjie Wu, Guoping Miu, Chenglin Chu

**Affiliations:** a Department of Orthopedics, Rudong People's Hospital Nantong 226400 Jiangsu China miuguoping@163.com; b Affiliated Rudong Hospital of Xinglin College, Nantong University 226007 Jiangsu China; c School of Materials Science and Engineering, Southeast University Nanjing 211189 China clchu@seu.edu.cn; d Jiangsu Key Laboratory for Advanced Metallic Materials, Southeast University Nanjing 211189 China

## Abstract

The insufficient osteogenesis of magnesium phosphate cement (MPC) limits its biomedical application. It is of great significance to develop a bioactive MPC with osteogenic performance. In this study, an injectable MPC was reinforced by the incorporation of a near infrared (NIR)-responsive nanocontainer, which was based on simvastatin (SIM)-loaded mesoporous silica nanoparticles (MSNs) modified with a polydopamine (PDA) bilayer, named SMP. In addition, chitosan (CHI) was introduced into MPC (K-struvite) to enhance its mechanical properties and cytocompatibility. The results showed that nanocontainer-incorporated MPC possessed a prolonged setting time, almost neutral pH, excellent injectability, and enhanced compressive strength. Immersion tests indicated that SMP-CHI MPC could suppress rapid degradation. Based on its physicochemical features, the SMP-CHI MPC had good biocompatibility and osteogenesis properties, as shown *via in vitro* and *in vivo* experiments. These findings can provide a simple way to produce a multifunctional MPC with improved osteogenesis for further orthopedic applications.

## Abstract 2

In this article, we reported a specific magnesium phosphate bone cement which showed good physical, chemical properties, and excellent osteogenesis-promoting ability under near-infrared light intervention.

## Graphical abstract

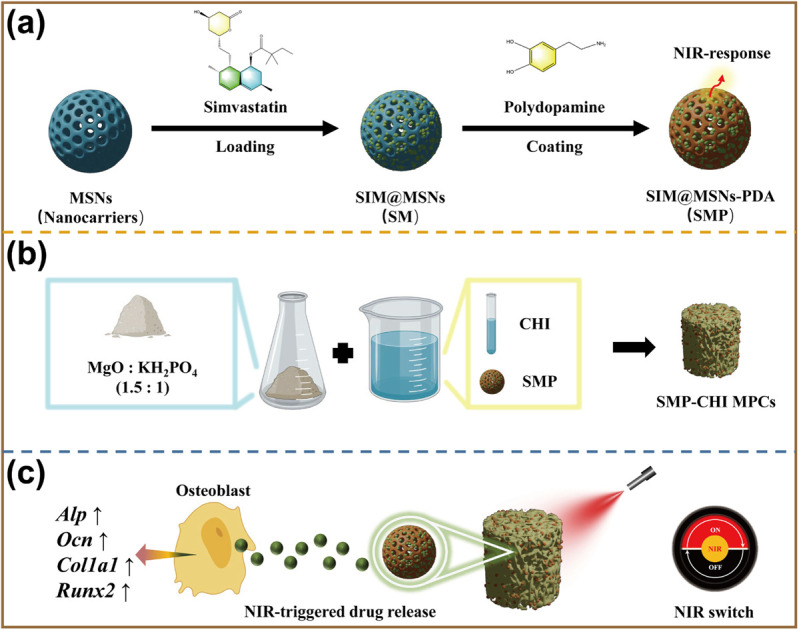

## 1. Introduction

Bone cement has wide applications in vertebral filling, joint prosthesis fixation, bone defect reconstruction, and other clinical issues. Nowadays, there are two bone cements commonly used in the clinic: polymethyl methacrylate and calcium phosphate bone cements, but these two types of cement have some defects like excessive heat release, lack of antibacterial activity, weak bone induction and insufficient mechanical properties.^1–4^ In recent years, increasing attention has been drawn to a new type of bone cement based on MgO and phosphate compounds (magnesium phosphate cement, MPC). The dissolved cations of magnesium oxide or phosphate react with phosphate anions to form an amorphous gel.^5–7^ This gel crystallizes around unreacted magnesium oxide or phosphate particles to form a core–shell solution and a durable material.

Compared with CPC, the strength of MPC is reflected in a shorter setting time, higher strength achieved earlier, more moderate pH value, and higher cohesion. The porosity and crystal density of the internal compounds of the MPC are similar to human cortical bone; therefore, as an implant, the MPC shows good tightness. In addition, the avoidance of the MPC of a faster degradation rate of the implant removes the need for surgery and secondary injury during bone healing .^8–14^

Although MPC materials have many advantages, their ability to promote bone formation in bone defect implants is still unsatisfactory. Statins were initially developed and applied to the treatment of cardiovascular diseases. At present, many studies show that statins have great application prospects in the field of bone regeneration. The main mechanism of simvastatin in promoting bone repair is to promote the expression of bone morphogenesis protein-2 (BMP-2) in osteoblasts, thereby promoting osteogenesis. In addition, simvastatin can promote the expression of vascular endothelial growth factor (VGEF), thus accelerating the rate of new bone formation.^15–17^

A local drug delivery system composed of nanomaterials and bone promoting drugs is currently a new strategy in the field of orthopedics. Due to the poor chemical stability and low solubility of simvastatin, encapsulating it in nanocarriers can help prolong drug release. Mesoporous silica nanospheres (MSNs) with mesoporous shells and internal hollow cavities have been used as nanostructure carriers for drug or reagent loading. The material has good biocompatibility, especially vascular compatibility, making it possible to inject through veins. In addition, adding various coatings to MSNs can endow them with certain physical and chemical response capabilities, making it possible to control drug release, such as response to light, temperature, and magnetic fields.^18–20^ To accurately control the leaching of simvastatin from nanocontainers, polydopamine (PDA) with rich amine and catechol binding was used for the surface modification of MSNs.^21–23^ PDA is a widely studied near-infrared absorption material with good biocompatibility. It does not cause significant biological toxicity at high doses and can be completely degraded in organisms. Due to its excellent photothermal exchange efficiency, it can improve the release efficiency of loaded drugs under near-infrared light intervention when used as a coating.^24–26^

Visible and ultraviolet light, due to their high light energy and low tissue penetration, can cause significant damage to biological tissues and organs, and it is difficult to turn on the switch of photothermal stimulation, resulting in a low drug release rate. Therefore, they are not suitable for practical *in vivo* applications.^27–30^ Compared to visible and ultraviolet light, the NIR region (650–900 nm) is also known as the “bio transparent window”.^31^ In this wavelength range, the scattering and absorption of light are minimized, resulting in much deeper penetration of near-infrared radiation into biological tissues than ultraviolet and visible light, thereby greatly increasing the drug release rate while minimizing damage to the biological organism.^32–34^

**Fig. 1:**
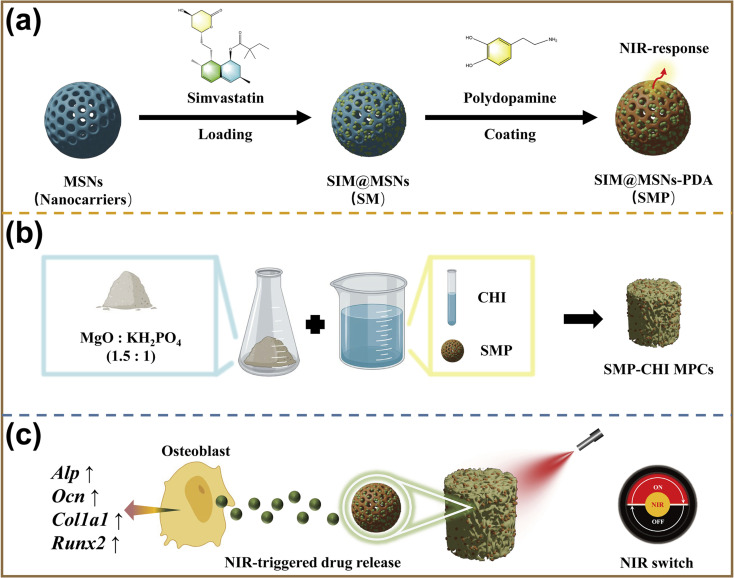
Schematic illustration of the experimental flow: (a) fabrication of SMP nanocontainers. (b) fabrication of SMP–CHI MPC. (c) NIR-triggered drug release and promotion of osteogenic differentiation.

In this study, we loaded simvastatin onto polydopamine-modified MSNs and subsequently incorporated them into the MPC which gives the MPC good mechanical properties and biocompatibility while having good bone-promoting ability under NIR intervention (Fig. 1).

## 2. Materials and methods

### 2.1 Materials

Magnesium oxide (MgO, *M*_w_: 40.3 g mol^−1^, 98.0%) purchased from Shanghai Macklin Biochemical Co., Ltd was first calcined for 4 h at 1600 °C to reduce activity and obtain dead-burnt magnesium oxide (d-MgO). Potassium dihydrogen phosphate (KH_2_PO_4_, *M*_w_: 136.09 g mol^−1^, ≥99.0%) was provided by Sinopharm Chemical Reagent Co., Ltd. Chitosan (CHI, *M*_w_: 100 kDa, deacetylation 80–90%) was obtained from Shanghai Yien Chemical Technology Co., Ltd. Simvastatin (SIM, *M*_w_: 418.57 g mol^−1^, ≥97%), tris(hydroxymethyl) aminomethane (tris, *M*_w_: 121.14 g mol^−1^, 99.9%), dopamine hydrochloride (*F*_w_: 189.64 g mol^−1^, 98%), ammonia solution (NH_3_ (aq), *F*_w_: 17.03 g mol^−1^, 25.0–28.0%), poly(ethylene glycol)-*block*-poly(propylene glycol)-*block*-poly(ethylene glycol) (Pluronic F127, *M*_n_: ∼12 600 g mol^−1^), hexadecyltrimethylammonium bromide (CTAB, *M*_w_: 364.45 g mol^−1^, ≥98%), and tetraethyl orthosilicate (TEOS, *F*_w_: 208.33 g mol^−1^, ≥28.4%) were purchased from Shanghai Aladdin Biochemical Technology Co., Ltd. These reagents were used in this study without purification.

### 2.2 Synthesis of simvastatin-loaded nanocontainers

A modified Stöber method was used to synthesize mesoporous silica nanoparticles (MSNs).^35^ Briefly, F127 (1 g mL^−1^) and CTAB (4.1 g mL^−1^) were mixed in an alcohol–water mixture (v/v = 86.2 mL/192 mL), and NH_3_ (aq, 22.4 mL) was then added. TEOS (3.86 mL) was quickly injected into this blend and stirred for another 15 hours. After centrifuge-wash-dry steps, the white precipitate was collected and calcined for 6 hours at 550 °C after 1 °C min^−1^ of heating. After that, pure MSNs were prepared.

For drug loading, simvastatin was directly embedded in mesoporous materials. In detail, as-synthesized MSNs (5 mg mL^−1^) were dispersed in simvastatin solution (1 mg mL^−1^) and later incubated for 1, 3, and 5 h, separately. After that, SIM-loaded MSNs (S@MSNs, SM) were gathered by centrifugation, washing, and drying under vacuum. Further, 5 mg mL^−1^ of Dopa·HCl was dissolved in Tris buffer (1.21 mg mL^−1^, pH 8.5) to prepare polydopamine solution, and SM (5 mg mL^−1^) were dispersed into 20 mL of this suspension under dark conditions for 1 h. After centrifugation and drying, polydopamine-modified SM (SMP) were collected.

### 2.3 Preparation of bioactive cements

d-MgO and KH_2_PO_4_ powders (∼75 μm particle sizes) were prepared by a ball milling method and further sieved. MPC powders were obtained by blending d-MgO and KH_2_PO_4_ in a mass ratio of 1 : 1.5 g. After that, these powders were added into cement liquid phases of ultrapure water, CHI and SMP-CHI solutions to prepare H_2_O MPC, CHI MPC, and SMP–CHI MPC, respectively. Herein, chitosan (2 mg mL^−1^) was dissolved in acetic acid (1 wt%), and the pH was adjusted to about 3.0, whereas 0.2 mg mL^−1^ of SMP was mixed with this CHI solution to prepare the SMP–CHI suspension. The powder to liquid ratios (PLRs) of H_2_O, CHI, and SMP–CHI cements were 5 : 1, 5 : 1.125, and 4 : 1, respectively. Herein, H_2_O MPC was selected as a control.

Cement powders and liquid phase were stirred to form a paste using a glass rod and later moved to cylindrical silicone molds (*φ* 9 × 10 mm^3^ for *in vitro* tests, *φ* 3 × 10 mm^3^ for *in vivo* assays). After curing, the MPCs were washed and cured in a 100% humidity incubator at 37 °C for one day to prevent a hydration reaction.

### 2.4 Materials characterization

The acid–base properties of the liquid phase were examined with a pH meter, as well as the MPCs after 24 h of immersion in phosphate-buffered saline (PBS, pH 7.4) in tubes. Optical images of the cements were captured by a Canon 5D Mark III SLR camera. Morphologies and elemental analyses for materials were evaluated using field-emission scanning electron microscopy (FE-SEM, Nova Nano SEM 450, USA) and energy-dispersive X-ray spectrometry (EDS). In addition, X-ray diffraction (XRD, Bruker D8-Discover, Germany), with a copper solid target (0.15° per s scanning rate, gathered from 5° to 65°), was used to detect the phase components of the cements. Transmission electron microscopy (TEM, Talos F200X S/TEM, USA) was used to measure the mesoporous architectures of the MSNs, SM, and SMP, and their particle sizes were analyzed using ImageJ. N_2_ adsorption/desorption isotherms of the nanoparticles were evaluated using a porosimeter system (Quantachrome Autosorb-IQ2, USA).

### 2.5 Setting time and exothermic reaction

The setting times of the cements were evaluated according to a standard Vicat apparatus. In detail, the paste-like MPCs were first transferred into silicone molds (*φ* 10 × 10 mm^3^). The setting time was recorded by mixing cement powders with liquid phases until the Vicat needle passed through the solidified cements within one millimeter. In addition, the infrared thermogram (UNI-T, UTi120s, China) was used to monitor variations in temperature of the cements during the preparation process. Every test for a specimen was repeated at least three times.

### 2.6 Injectability

To measure the injectability, cement pastes were put into syringes (2.5 mL). By pushing the plunger, the volumes of the remaining cements were recorded from the end of the fluid state to the unpropelled point. The injectability of the MPCs could be quantified by calculating the injection rate (%), as below:1
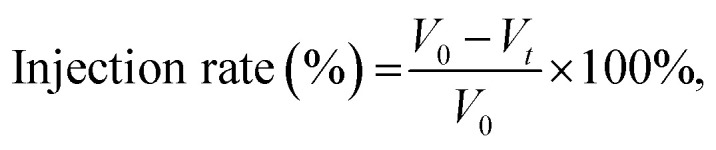
where *V*_0_ and *V*_*t*_ are the remaining volumes at the beginning and at time *t*.

### 2.7 Compressive strength and porosity

A universal testing machine XQY-II was used to measure the compressive strength of the samples. The loading rate was 1 mm min^−1^ in this work. According to Archimedes' principle, the porosities of the materials after 24 hours of curing were also characterized by an AR-300G electronic densimeter. The cements were immersed in ultrapure water until completely wetted, and their porosities (%) were calculated on the basis of formula (2):2
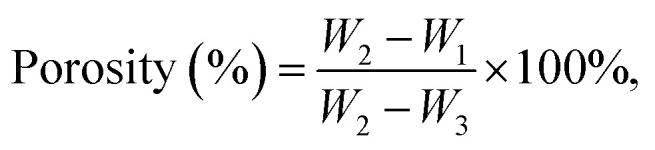
where *W*_1_ and *W*_2_ represent the mass of the material before and after madefaction, and the suspended mass of the immersed cement is denoted as *W*_3_.

### 2.8 Simvastatin loading and NIR-responsive release profiles

A dual-beam ultraviolet–visible spectrophotometer (UV–vis, TU-1901) provided by Beijing Puxi General Instrument Co., Ltd was used to evaluate the absorbance of simvastatin at 246 nm. The amount of drug loading was calculated by subtraction of dissociative simvastatin in the washing from the initial quantity. Thus, the drug loading efficiency (DLE, %) could be calculated according to eqn (3):3
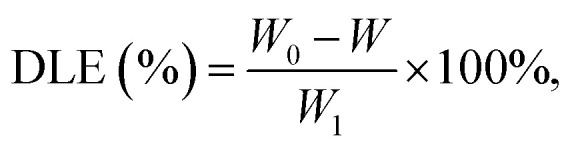
where *W*_0_ and *W* represent initial and free masses of simvastatin, and *W*_1_ is the total weight of SM.

To measure the NIR-responsive controlled release of simvastatin from SM and SMP nanoparticles, 808 nm near-infrared lasers with power densities of 0.5, 1.0, and 2.0 W cm^−2^ were adopted to shine on nanoparticle materials incubated in PBS. The cumulative drug release rate (DRR, %) was calculated with eqn (4):4
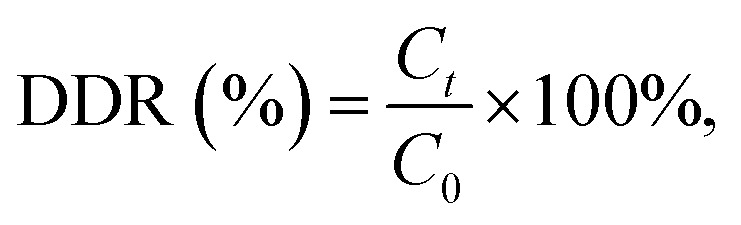
where *C*_*t*_ and *C*_0_ are the concentrations of leached and initially loaded simvastatin at time *t*.

### 2.9 *In vitro* degradation behavior

The degradation of the cements was measured by immersion in PBS (pH 7.4) at 37 °C for 14 days. In brief, the initial weights of the different MPCs kept in 100% humidity for one day at 37 °C were denoted as *M*_0_. Then, they were immersed in PBS at a solid to liquid ratio of 20 mg to 1 mL. The corroded samples were dislodged and dried in a 40 °C ventilated drying cabinet for one day, and their weights were denoted as *M*_1_. At the same time, pH values were also recorded. The weight loss rate (WLR, %) of the cement was calculated on the basis of formula (5):5
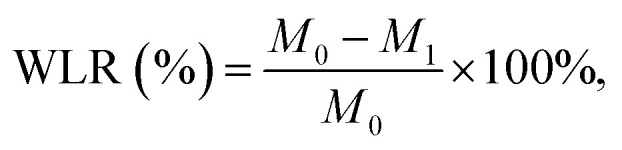


The morphologies and compositional elements of the cements after degradation were evaluated by SEM and EDS, and their components were detected *via* XRD.

### 2.10 Preparation of cell culture extract

A mixture of bone cement and complete cell culture medium (DMEM + 10% FBS) at a 0.2 g mL^−1^ ratio was extracted in a cell incubator (37 °C, 5% CO_2_) for 24 hours. Then, the extract was processed for subsequent cell experiments by (1) centrifuging at 4000 rpm for 10 minutes and collecting the supernatant; (2) 0.22 μM membrane filtration of the supernatant.^36^

### 2.11 Cell proliferation detection

We used the CCK-8 method to detect the proliferation ability of C3H10 cells. The cells were cultured in 96-well plates with a complete medium at a density of 1 × 10^3^; the original medium was discarded after 1 day, and the extract was added; on days 1, 3 and 5, we added 10 μL of CCK-8 test liquid into each well, and after incubating in a cell incubator for 1 hour, we could record the absorbance under a microplate reader (450 nm).

### 2.12 Apoptosis

The C3H10 cells were cultured in a 6-well plate at a density of 1 × 10^6^. We discarded the original medium when the cell density reached 80%. Then, we followed given steps to detect the apoptosis level: (1) wash the cells twice with cold PBS and later resuspend the cells in 1× binding buffer at a concentration of 1 × 10^6^ cells per mL; (2) transfer 100 μL of the solution (1 × 10^5^ cells) to a 5 mL culture tube; (3) add 5 μL of FITC Annexin V and 5 μL of PI; (4) gently vortex the cells and incubate for 15 min at RT (25 °C) in the dark; (5) add 400 μL of 1× binding buffer to each tube. Analyze by flow cytometry within 1 h.

### 2.13 Subacute toxicity experiment *in vivo*

Bone cement and physiological saline were mixed at a 0.2 g mL^−1^ ratio and extracted in a cell incubator (37 °C, 5% CO_2_) for 24 hours. Then, given steps were followed for the subsequent toxicity experiment: (1) centrifuge at 4000 rpm for 10 minutes and collect the supernatant; (2) 0.22 μM membrane filtration of the supernatant. Subsequently, the extract was injected into a mouse body through the abdominal cavity at a ratio of 50 mL kg^−1^.

The mice (C57BL/6) were divided into 4 groups, each with 5 male and 5 female mice. The weights of the mice were measured before injection, 1 week after injection, and 2 weeks after injection. One mouse in each group was randomly selected to take the viscera (heart, liver, spleen, lungs, and kidneys) for pathological sectioning. Four mice in each group were randomly selected to take blood from their eyeballs, and the expression level of the serum inflammatory factors (IL-6, IL-1β) was detected.

### 2.14 Osteogenic induction

The configuration of osteogenic induction fluid followed the addition of 500 μL of β-glycerophosphate (10 mM), 50 μL of dexamethasone (10 nM) and 100 μL of ascorbic acid (50 μg mL^−1^) into 50 mL of extract. We discarded the original medium when the cell density reached 80%, cleaned the cells using PBS, and later added an induced medium and replaced it with a frequency of 2 days.

### 2.15 ALP/ARS staining and quantification analysis

Cells were cultured in a 6-well plate at a density of 2 × 10^6^. We discarded the original medium before staining and cleaned the cells twice using PBS, and then, we used the BCA (bicinchoninic acid) method to prepare protein samples. The ALP/ARS staining was analyzed quantitatively under a microplate reader with 562/405 nm absorbance.

An ALP activity test followed these steps: (1) cells were cultured in a 96-well plate at a density of 1 × 10^3^; (2) the original medium was discarded before staining, and the cells were cleaned twice using PBS; (3) lysis solution was added, and the supernatant was collected after centrifugation, mixed with the detection reagent and incubated for 30 min at room temperature (dark environment); (4) the absorbance was measured at 405 nm under a microplate reader.

### 2.16 Real-time PCR

Total RNA was extracted from the cells using the TRIzol method and reverse transcribed into cDNA. Through qPCR experiments, the expression levels of osteogenic differentiation characteristic genes and muscle atrophy genes were detected in myotube cells induced by C3H10 and C2C12 cultured in extract solution. The primers involved are shown in Table 1.

**Table 1:** RT-PCR primer sequences

Genes	Forward (5′–3′)	Reverse (5′–3′)
*Gapdh*	TTCCAGGAGCGAGACCCCACTA	GGGCGGAGATGATGACCCTTTT
*Ocn*	GGTAGTGAACAGACTCCGGC	GGCGGTCTTCAAGCCATACT
*Alp*	TCCGTGGGCATTGTGACTAC	TGGTGGCATCTCGTTATCCG
*Runx2*	GACTGTGGTTACCGTCATGGC	ACTTGGTTTTTCATAACAGCGGA
*Col1a1*	TTCTGCTGCTAATGTTCTTGACC	GGGATGAAGTATTGTGTCTTGGG

### 2.17 Statistical analysis

The data are expressed as mean ± standard deviation. Statistical differences between three or more groups were analyzed by one-way analysis of variance (ANOVA) and a Bonferroni post hoc test. The significance of the difference between the two groups was determined through an unpaired Student's test. Statistical analysis was conducted using SPSS software 19.0. *P* < 0.05 is considered statistically significant.

## 3. Results and discussion

### 3.1 Characterization of the prepared cements

**Fig. 2:**
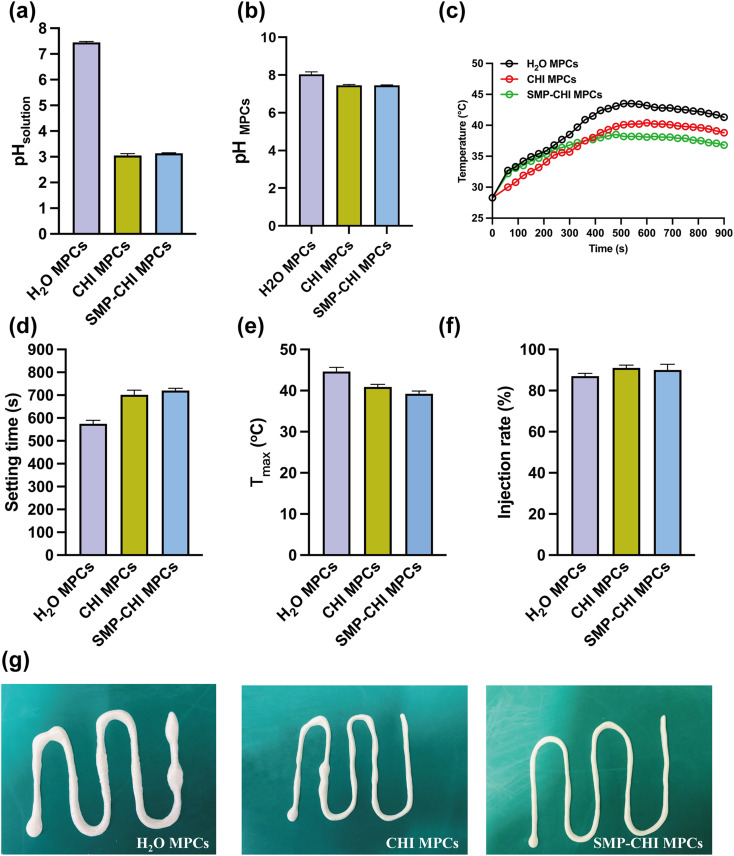
(a) pH of liquid phase for cements. (b) pH of the prepared cements. (c) Temperature variation of MPCs in fabrication. (d) Setting time of the cements. (e) Maximum temperature of the cements in an exothermic reaction. (f) Injection rate of MPCs. (g) Representative photographs of injectability for cements.

In the preparation process, the hydration reaction between d-MgO and KH_2_PO_4_ results in an alkaline environment. It is crucial to use an acidic liquid to adjust the pH of the cements. The pH of CHI and SMP–CHI MPCs were 3.05 and 3.14, which were lower than that of H_2_O MPC (pH 7.45), as shown in Fig. 2a. After solidification (Fig. 2b), the control group showed alkalinity (pH 8.05), and yet the pH of both the other two cements were about 7.45. Temperature changes for the MPCs during solidification were monitored within 15 min, as shown in Fig. 2c. The heat that is being released exhibited a tendency to first rise and further diminish in a stepwise manner.

### 3.2 Setting time and exothermic analysis

As shown in Fig. 2d, it was obvious that the setting times of the cements were prolonged with the addition of chitosan and SMP. The H_2_O MPC exhibited a rapid hydration reaction within a setting time of 575 s. However, the curing times of the MPCs were extended to 702 s and 720 s after the incorporation of CHI and SMP–CHI materials. Besides, the maximum temperature (*T*_max_) during the curing process of the control group was 44.6 °C, but decreased to 40.9 °C and 39.2 °C after adding CHI and SMP–CHI, as shown in Fig. 2e.

### 3.3 Injectability

In Fig. 2f, only 87% of the H_2_O MPC could be extracted from the syringe, but the injection rates of the CHI and SMP–CHI MPCs increased to 91% and 90%, respectively, indicating good injectability of the cements due to the incorporation of chitosan and SMP. Representative photographs (Fig. 2g) of injectable MPCs were also taken. It could be seen that the injectability of the SMP–CHI MPC was enhanced compared with the control and CHI groups.

### 3.4 Surface characterization and compositional analysis

**Fig. 3:**
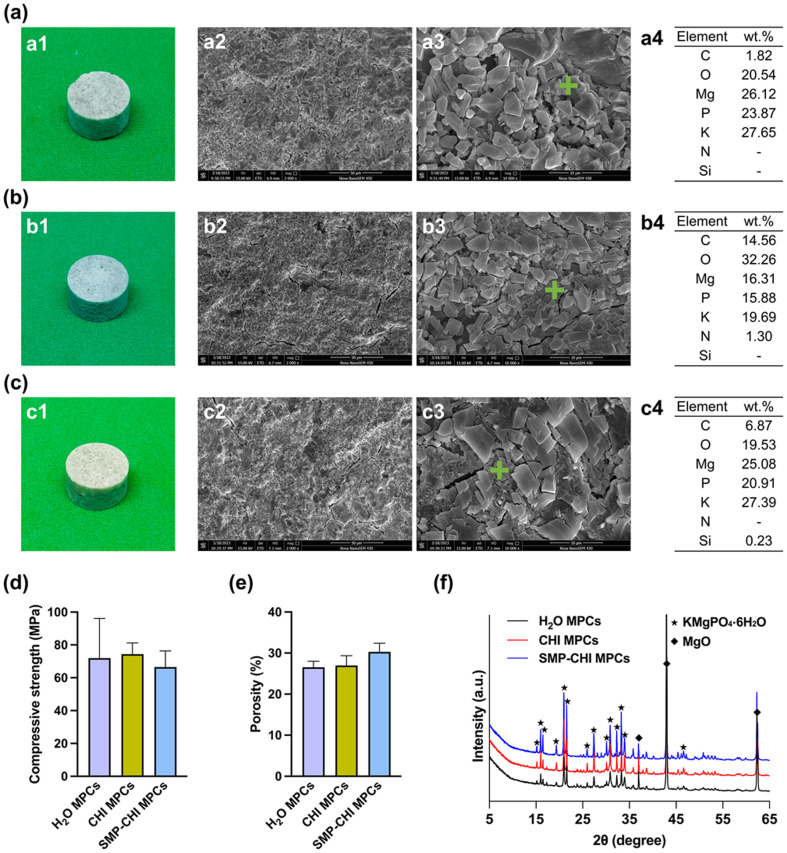
Optical images, morphologies, and EDS analyses of (a) H_2_O MPC, (b) CHI MPC, and (c) SMP–CHI MPC. (d) Compressive strength of the cements. (e) Porosity of MPCs. (f) XRD of different samples.

The morphologies and compositional elements of the cements were evaluated and are shown in Fig. 3a–c. From the optical images (Fig. 3(a1)–(c1)), it could be seen that the surfaces of the CHI and SMP–CHI MPCs were denser than that of the control group. In addition, the morphology of the H_2_O cement exhibited some phosphate crystals with many cracks, but they were well sealed with polymers and nanoparticles on the CHI and SMP–CHI MPCs. In the EDS analysis, Si element was detected on the surface of the SMP–CHI MPC, illustrating the successful incorporation of SMP nanocontainers into the bare cements. In XRD patterns (Fig. 3f), the phase composition of the materials was detected and showed the typical absorbance peaks of KMgPO_4_·6H_2_O and MgO.

### 3.5 Compressive strength and porosity

As shown in Fig. 3d, the H_2_O MPC exhibited a compressive strength of 71.97 MPa, and that of the CHI MPC was 1.03-fold stronger than that of the control. However, the strength of the SMP–CHI MPC (66.55 MPa) decreased due to the incorporation of nanocontainers. In Fig. 3e, the porosities of the H_2_O and CHI MPCs were almost the same, close to ∼26%, and yet that of the SMP–CHI group was about 30.28%. Therefore, we could infer that the decline in compressive strength of the SMP–CHI MPC was because of the increasing porosity.

### 3.6 Characterization of nanoparticles

**Fig. 4:**
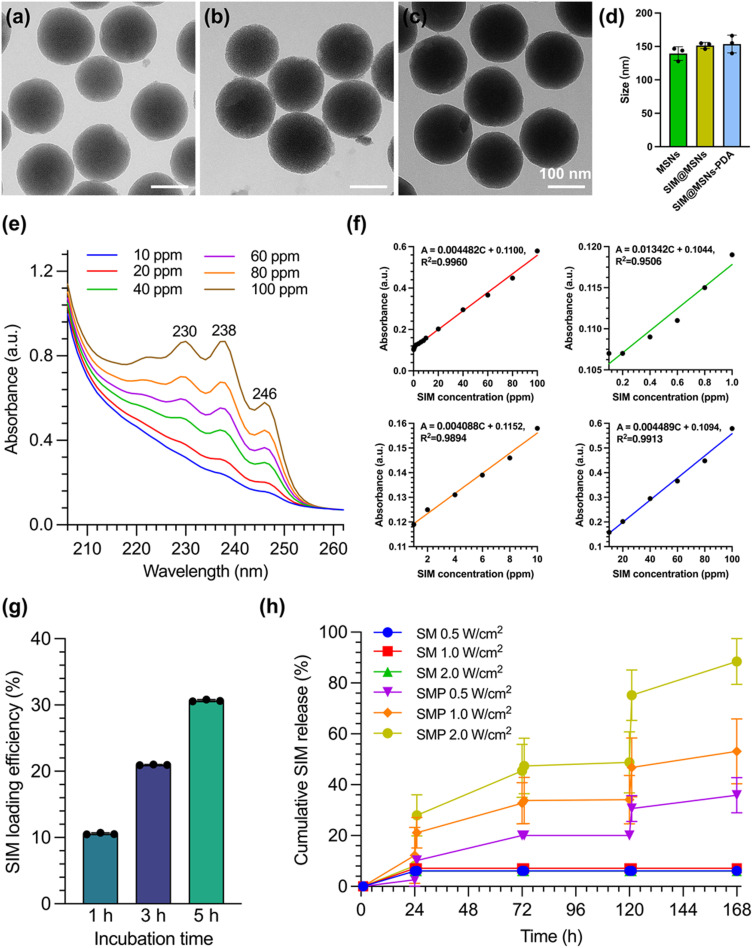
TEM of (a) MSNs, (b) SM, and (c) SMP nanoparticles. (d) Particle sizes. (e) UV–vis plots of the simvastatin dispersion with different concentrations. (f) Standard curve of simvastatin concentration *vs.* absorbance *via* UV–vis. (g) Drug loading efficiency *vs.* incubation time. (h) Simvastatin release profile from SM and SMP under 808 nm NIR irradiation with different densities of 0.5, 1.0, and 2.0 W cm^−2^ in PBS at pH 7.4.

In this study, MSN nanoparticles were synthesized on the basis of the modified Stöber method. In Fig. 4a, many well-ordered mesoporous pores extended through the interior of the nanoparticles. After encapsulation of simvastatin in Fig. 4b, the architectures of mesopores were almost filled with drugs. In Fig. 4c, a crude layer of polydopamine was coated on the outmost shell of the MSNs. In addition, the average particle size of SMP (153.61 nm) in Fig. 4d was larger than that of the MSN (139.38 nm) and SM (151.46 nm) materials, from which it could be inferred that the PDA layer was about 14.23 nm. In Fig. S1,† the surface area of the MSNs (220.61 ± 10.51 m^2^ g^−1^) decreased after simvastatin encapsulation (95.20 ± 3.63 m^2^ g^−1^), and they were further reduced to 34.27 ± 4.72 m^2^ g^−1^ after PDA coating. These results confirmed the successful preparation of SMP nanocontainers.

### 3.7 Drug loading and release profiles

In UV–vis spectra (Fig. 4e), typical absorbance peaks of simvastatin were confirmed at wavelengths of 230, 238, and 246 nm. The extract of simvastatin exhibited a positive correlation between concentration and absorbance, indicating good dispersibility, and their relationship is shown in Fig. 4f. The SIM-loaded MSNs showed time-dependent drug loading behavior, as shown in Fig. 4g. Cumulative amounts of SIM loading increased following an increase in incubation time from 1 to 5 h. Especially after 5 h of incubation, the treated SM possessed a maximum drug loading efficiency of 30.67%. To measure the NIR-responsive release of simvastatin from SM and SMP materials, they were irradiated by 808 nm NIR lasers with different power densities of 0.5, 1.0, and 2.0 W cm^−2^ for 10 min at time points of 1, 3, 5, and 7 days. In Fig. 4h, the SM showed no obvious stimuli-responsive characteristics within the entire period of 7 days. However, the release profile of SMP was accelerated with increasing laser power density. This demonstrated that the release of simvastatin from the nanocontainers could be controlled under the conditions of NIR irradiation.

### 3.8 Degradation behavior

**Fig. 5:**
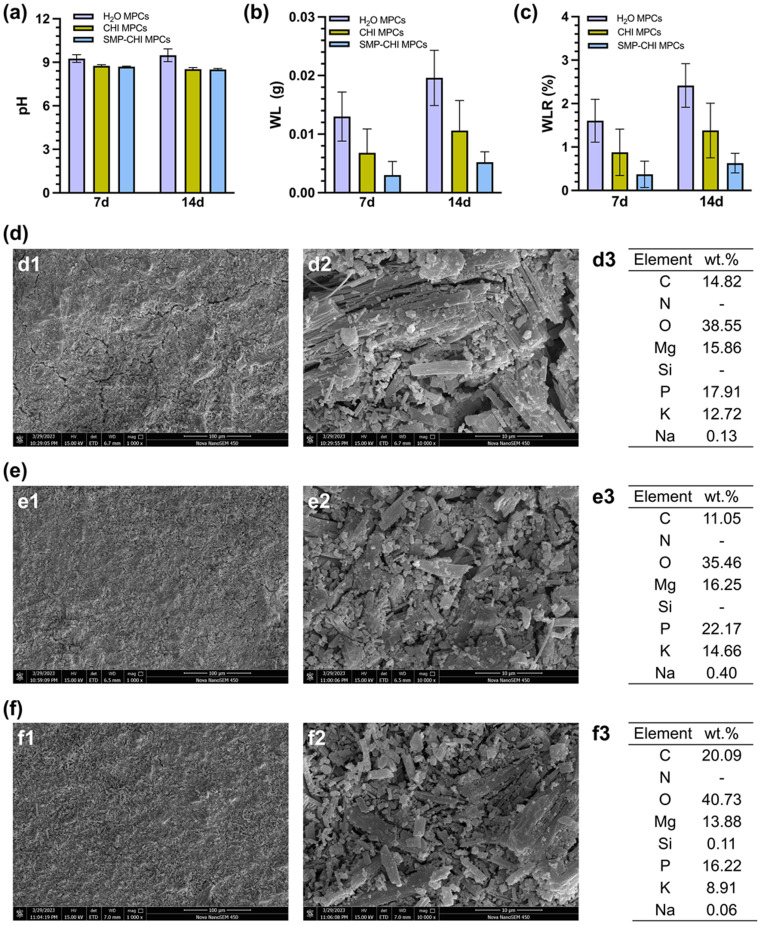
(a) pH, (b) weight loss, and (c) weight loss rate of different cements after immersion for 14 d in PBS. Morphologies and EDS analyses of (d) H_2_O MPC, (e) CHI MPC, and (f) SMP–CHI MPC.

To measure the degradation behavior of the cements, they were immersed in PBS (pH 7.4) for 14 days. In Fig. 5a, the pH values of the H_2_O MPC were 9.26 and 9.49 after 1 and 2 weeks of immersion, respectively, but those of the CHI and SMP–CHI MPCs were almost the same and were around 8.7 and 8.5, respectively, at the same time point. The incorporation of chitosan and SMP nanocontainers showed an inhibitory effect on weight loss and weight loss rate of the cements, as shown in Fig. 5b and c. Especially after immersion for 14 days, the WLR of the control group was 2.42%, but it decreased to 1.38% and 0.63%, respectively. The surface morphologies and elements of corroded samples were measured using SEM and EDS, as shown in Fig. 5d–f. It could be seen that the H_2_O MPC were severely corroded within 14 days of immersion, and yet the CHI and SMP–CHI cements were slightly damaged. EDS analyses illustrated that the SMP nanocontainers were still present in CHI and SMP–CHI cements.

### 3.9 Cell proliferation and apoptosis

**Fig. 6:**
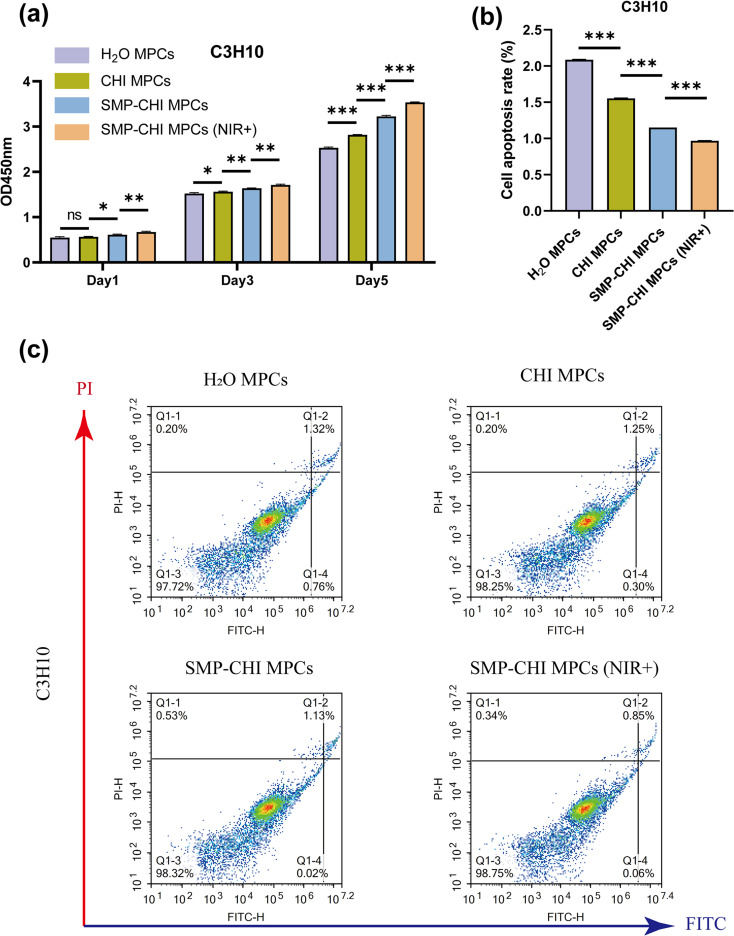
(a) Proliferation level of C3H10 cells at 1, 3, and 5 days among the 4 groups. (b) Cell apoptosis rate among the 4 groups. (c) Scatterplot figures under flow cytometry, which indicate the apoptosis level of C3H10 cells. *p < 0.05, **p < 0.01, and ***p < 0.001, ns: no statistical significance.

We evaluated the biocompatibility of H_2_O MPC, CHI MPC, SMP–CHI MPC, and SMP–CHI MPC (NIR+) groups of bone cement on C3H10 cells using CCK-8 and apoptosis levels (using flow cytometry). Fig. 6a shows that after culturing C3H10 cells with bone cement extract for 1, 3 and 5 days, it was found that there was no significant cytotoxicity in the four intervention groups, and the proliferation level of the cells showed an increasing trend with the increase in culture time. In addition, Fig. 6b and c showed that after culturing the cells with bone cement extract in the four groups, there was no significant proapoptotic effect (cell apoptosis rate < 10%). CCK-8 and flow cytometry apoptosis detection tests were performed, and all indicate that SMP–CHI MPC (NIR+) has good cell compatibility.

### 3.10 Subacute toxicity test *in vivo*

The safety of implants depends on an evaluation of cell proliferation and apoptosis ability through *in vitro* experiments and observation of the changes in animal tissues and organs through *in vivo* experiments to further measure their biocompatibility and toxicity.

**Fig. 7:**
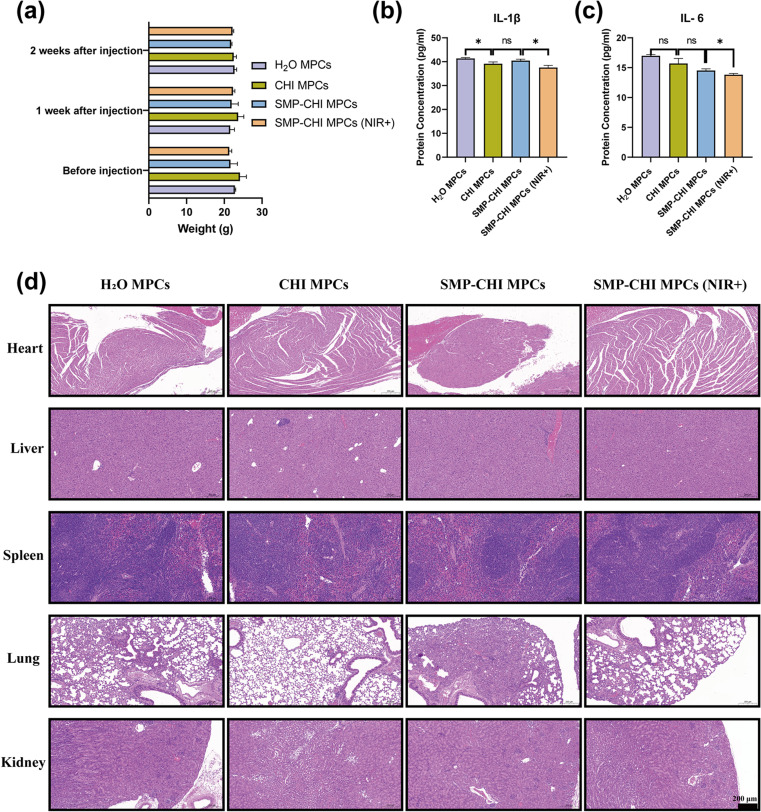
Biosafety and safe implantability of SMP–CHI MPC. (a) Changes in body weight of the C57BL/6 mice before and after bone cement treatment. (b and c) Expression levels of inflammatory factors in the serum of C57BL/6 mice treated with bone cement. (d) H&E staining of major visceral organs of C57BL/6 mice treated with bone cement. *p < 0.05, **p < 0.01, and ***p < 0.001, ns: no statistical significance.

In this study, we used physiological saline to extract bone cement and further injected the extract into the tail veins of mice. Before injection and 1 week and 2 weeks after injection, there was no significant statistical difference in body weight among the four groups of mice (Fig. 7a), indicating that the bone cement did not show a significant immune rejection reaction in mice. To further evaluate the subacute toxicity of the bone cement extract on the mice, we first detected IL-6 and IL-1β proteins related to the immune response in mouse serum by ELISA 2 weeks after injection, and the results showed that compared to the other 3 groups, the expression levels of the SMP–CHI MPC (NIR+) group of the two mentioned inflammatory factors were lowest (Fig. 7b and c). Moreover, HE staining of the organs of each group of mice showed no significant toxic effect on tissues after injection of bone cement extract (Fig. 7d).

### 3.11 Effect of bone cement on osteogenic differentiation

**Fig. 8:**
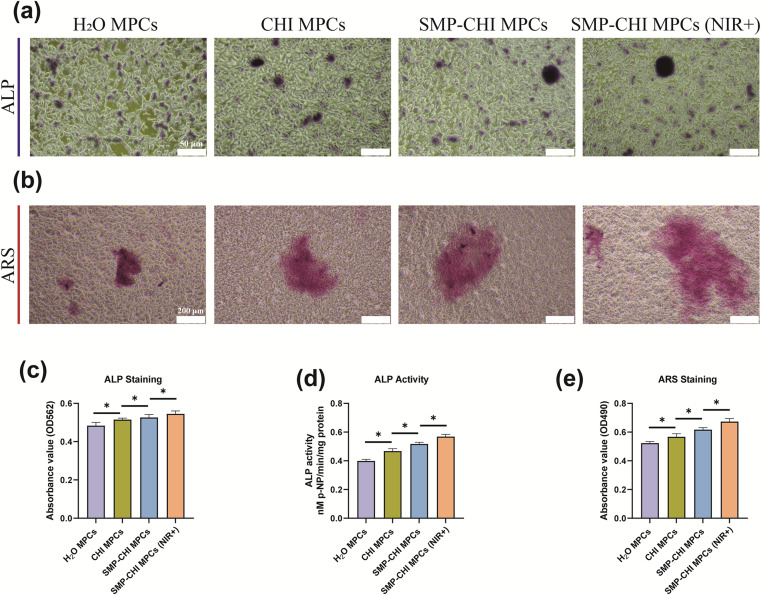
(a) ALP staining result under a light microscope at 7 days among the 4 groups. (b) ARS staining result under a light microscope at 14 days among the 4 groups. (c) Quantified analysis result of ALP among the 4 groups. (d) ALP activity result among the 4 groups; (e) quantified analysis result of ARS among the 4 groups. *p < 0.05, **p < 0.01, and ***p < 0.001, ns: no statistical significance.

**Fig. 9:**
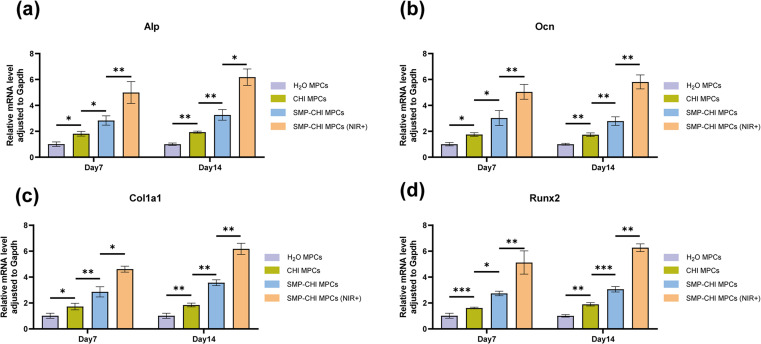
Relative MRNA expression of some osteogenic differentiation genes, *Alp*, *Ocn*, *Col1a1* and *Runx2* of C3H10 cells cultured on the various samples for 7 and 14 days. *p < 0.05, **p < 0.01 and ***p < 0.001, ns: no statistical significance.

For implants in the field of orthopaedics, in addition to exploring their toxicity to cells, the most important thing is to pay attention to their impact on osteogenic ability. In this study, the experimental results of Fig. 8a, c and d showed that when bone cement induced cells reached the 7th day, ALP staining, ALP quantification, and ALP activity experiments, which can represent the early osteogenic ability of cells, showed that the bone cement had excellent performance in inducing bone formation. The SMP–CHI MPC showed better osteogenic ability than H_2_O or CHI MPCs, which was stronger after near-infrared irradiation of the SMP–CHI MPC. When bone cement induced cells reached the 14th day, ARS staining and corresponding quantitative experiments showed that the SMP–CHI MPC (NIR+) still had excellent osteogenic performance compared to the other groups with regard to mineral deposition during late osteogenesis (Fig. 8b and e). Fig. 9 shows that whether in the early stage (7 days) or late stage (14 days) of osteogenesis induction, the expression levels of mRNA that can be used to represent the osteogenesis ability in the SMP CHI MPC (NIR+) group: *Alp*, *Ocn*, *Col1a1* and *Runx2* are significantly higher than those in the other three groups, which indicates that under the intervention of near-infrared light, the release rate of simvastatin loaded in nanoparticles is accelerated. It has better promoting and inducing effects on bone formation.

## 4. Conclusions

In summary, we constructed a type of SMP–CHI MPC bone cement based on MgO and phosphate compounds. Compared with the control group, the SMP–CHI MPC exhibited better physical and chemical properties, lower biological toxicity, and better bone-promoting properties. Moreover, the medication release rate accelerated, and osteoinduction ability improved obviously under the intervention of short-range infrared. Although our studies have some limitations, such as the lack of an osteogenesis ability experiment *in vivo*, the SMP–CHI MPC has promising clinical applications.

## Ethical approval

All institutional and national guidelines for the care and use of laboratory animals were followed. *In vivo* experiments were conducted in accordance with the Chinese Animal Experimentation Law and approved by the Ethics Committee of Rudong People's Hospital (ethical number: 2022RDHGZRDWLS-00023).

## Author contributions

BW was involved in conceptualization, investigation, formal analysis and writing – original draft. YBZ was involved in investigation, formal analysis and writing – original draft. YYL and JYY were involved in investigation and formal analysis. SJW was involved in formal analysis. GPM was involved in methodology, supervision and writing – review and editing. CLC was involved in conceptualization, supervision, funding acquisition and writing – review and editing.

## Conflicts of interest

The authors declare that they have no conflict of interest.

## Supplementary Material

RA-014-D4RA01079E-s001
