# Supplementary material for: NIR-responsive magnesium phosphate cement loaded with simvastatin-nanoparticles with biocompatibility and osteogenesis ability
Source: RSC Adv. 2024 Apr 29;14(20):13958–71. doi: 10.1039/d4ra01079e (PMC11056825; doi:10.1039/d4ra01079e)
Supplement: RA-014-D4RA01079E-s001 [file RA-014-D4RA01079E-s001.pdf]

Electronic Supplementary Material (ESI) for RSC Advances.
This journal is © The Royal Society of Chemistry 2024

NIR-responsive magnesium phosphate cement loaded simvastatin- nanoparticles with biocompatibility and osteogenesis ability

Bin Wang[a,1], Yanbin Zhao [b,c,1], Yangyang Li [b,c], Junyan Yao [b,c], Shunjie Wu [a], Guoping Miu[a,*], Chenglin Chu [b,c,*]

[a] *Department of Orthopedics, Rudong People's Hospital, Nantong, 226400, Jiangsu, China*

[b] *School of Materials Science and Engineering, Southeast University, Nanjing, 211189, China*

[c] *Jiangsu Key Laboratory for Advanced Metallic Materials, Southeast University, Nanjing, 211189, China*

[*] Corresponding author.

Email address: miuguoping@163.com (G.P. Miu), clchu@seu.edu.cn (C.L. Chu),

[1] Bin Wang and Yanbin Zhao contributed equally to this article.

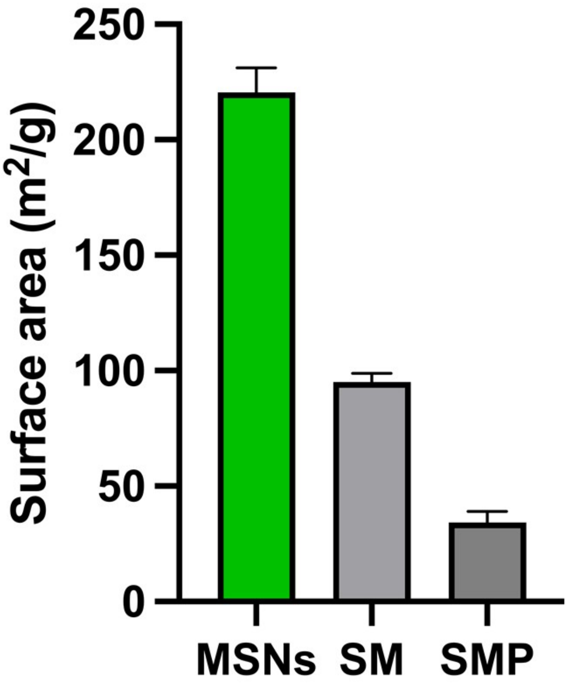

Fig. S1 Surface areas of MSNs, SM, and SMP nanoparticles.
